# Identification of allosteric disulfides from labile bonds in X-ray structures

**DOI:** 10.1098/rsos.171058

**Published:** 2018-02-07

**Authors:** Aster E. Pijning, Joyce Chiu, Reichelle X. Yeo, Jason W. H. Wong, Philip J. Hogg

**Affiliations:** 1 The Centenary Institute, Camperdown, New South Wales 2050, Australia; 2 National Health and Medical Research Council Clinical Trials Centre, University of Sydney, Sydney, New South Wales 2006, Australia; 3 Prince of Wales Clinical School and Lowy Cancer Research Centre, UNSW Sydney, Sydney, New South Wales 2052, Australia

**Keywords:** allosteric disulfides, redox, coagulation, complement, oxygen-sensing

## Abstract

Protein disulfide bonds link pairs of cysteine sulfur atoms and are either structural or functional motifs. The allosteric disulfides control the function of the protein in which they reside when cleaved or formed. Here, we identify potential allosteric disulfides in all Protein Data Bank X-ray structures from bonds that are present in some molecules of a protein crystal but absent in others, or present in some structures of a protein but absent in others. We reasoned that the labile nature of these disulfides signifies a propensity for cleavage and so possible allosteric regulation of the protein in which the bond resides. A total of 511 labile disulfide bonds were identified. The labile disulfides are more stressed than the average bond, being characterized by high average torsional strain and stretching of the sulfur–sulfur bond and neighbouring bond angles. This pre-stress likely underpins their susceptibility to cleavage. The coagulation, complement and oxygen-sensing hypoxia inducible factor-1 pathways, which are known or have been suggested to be regulated by allosteric disulfides, are enriched in proteins containing labile disulfides. The identification of labile disulfide bonds will facilitate the study of this post-translational modification.

## 1. Introduction

Allosteric disulfide bonds are defined by their ability to affect the functioning of the protein in which the bond resides. Reduction or oxidation of allosteric disulfide bonds leads to conformational transitions in the residing protein, that result in a change in either ligand binding, enzyme activity, proteolysis or oligomerization of the protein [1]. The allosteric bonds are cleaved or formed by oxidoreductases of the thioredoxin family or by intra- or inter-molecular thiol–disulfide exchange. Over 30 examples of allosteric disulfides have been described. The extent to which biological processes are controlled by allosteric disulfides has not yet been fully elucidated. However, certain processes are clearly regulated by this form of protein control. In humans, thrombosis and haemostasis is an example of a system that is regulated by allosteric disulfides [2]. Disease processes regulated by allosteric disulfide bonds include cancer [3] and viral infection [4]. Clinical relevance lies in the fact that these disulfide bonds can be targeted with inhibitors of certain factors that cleave the bonds, such as protein disulfide isomerase (PDI). Small molecule PDI inhibitors are being developed [5] and a first generation molecule is currently being tested as an anti-thrombotic in a Phase II cancer clinical trial [6].

Studies of the biophysical properties of allosteric disulfides have led to the recognition of defining features of these bonds. Firstly, a conformational signature for allosteric disulfides has been identified based on the sign of the five dihedral angles which define the cystine residue [7]. There are 20 different disulfide bond configurations based on this classification and 3 of the 20 are emerging as allosteric configurations: the –RHstaple, –LHhook and –/+RHhook bonds. Secondly, the –RHstaple and –/+RHhook bonds are more stressed than the other 18 disulfide types [8], which is primarily due to stretching of the sulfur–sulfur bond and neighbouring bond angles. Stretching of sulfur–sulfur bonds is known to accelerate their cleavage [9–12], so the pre-stress of the –RHstaple and –/+RHhook configurations is very likely important for their reduction and has probably influenced their evolution as allosteric bonds.

The three allosteric configurations constitute approximately 20% of all disulfide bonds in X-ray structures in the Protein Data Bank (PDB) [7]. While bond configuration has proved useful for identifying allosteric disulfides in proteins, it is likely that many, if not most, bonds with allosteric configurations will not have a functional role. Additional methods are needed to identify this post-translational modification. Here, we identify 511 labile disulfide bonds in PDB X-ray structures from bonds that are present in some molecules of a protein crystal or in some structures of a protein, but absent in others. A notable feature of the labile bonds is their pre-stress that likely underlies their facile nature. Biological pathways enriched in proteins containing labile disulfides are the complement and coagulation cascades and cytoplasmic oxygen-sensing hypoxia inducible factor-1 (HIF-1) system. Potential allosteric disulfide bonds in these pathways are presented.

## 2. Methods

All X-ray structures released in the PDB as of June 2017 were assembled. The list was culled to exclude all structures with a resolution >2.5 Å. Structures that had been prepared and crystallized in the presence of dithiothreitol or any other reducing agent were removed from the analysis.

To identify missing disulfide bonds, each PDB chain was first mapped to a corresponding UniProtKB accession and protein sequence using the PDBSWS tool [13]. Subsequently for each UniProtKB accession, a list of corresponding PDB chains and disulfide bonds present in any of these PDB chains were recorded. Disulfide bonds in structures were determined by the presence of an SSBOND line in the PDB file. Finally, for each disulfide bond that has now been associated with each UniProtKB protein, all PDB chains mapped to this corresponding UniProtKB accession were analysed to determine whether this disulfide bond is present or missing. If the disulfide bond is missing within a particular PDB chain, that structure was further analysed to establish whether the bond is missing due to a truncated or mutated PDB chain protein sequence. The annotation of each disulfide bond was performed as described previously [7]. A schematic diagram illustrating the analysis is shown in electronic supplementary material, figure S1.

For the analysis of disulfide bond features, a list of culled disulfide bonds from all PDB structures was used. To define the set of culled disulfide bonds, the PISCES PDB culling server was used with a cut-off of 90% homology, maximum resolution of 2.5 and *R* value of 1.0. Subcellular localization of proteins were obtained from the UniProtKB database. Pathways enriched in human proteins containing labile disulfide bonds were identified using the DAVID Functional Annotation Tool and KEGG pathway analysis [14,15].

A Python script was developed implementing the above analysis and can be downloaded from https://github.com/jwon7011/missing_disulfide.

## 3. Results

A total of 1361 unique labile disulfide bonds were identified from the PDB as of June 2017. The reference dataset consisted of all 14 033 unique disulfide bonds in the PDB (electronic supplementary material, table S1). To eliminate poorly defined or erroneous bonds, criteria of a structure resolution <2.5 Å and sulfur–sulfur distances < 10% from the disulfide bond equilibrium length of 2.038 Å [8] were applied. The datasets were refined to present each unique disulfide bond as a single entry (electronic supplementary material, table S2). The final list contains 511 labile disulfides and 13 030 total disulfides.

In X-ray crystallography, the B factor is a measure of the degree to which the electron density of an atom is dispersed. To ensure that the missing disulfide bonds that we detected were not due to uncertainty in the position of cysteines, we compared the average B factor of disulfide-bonded cysteines with those of matched missing disulfide bonds. The B factor for each atom of each cysteine involved in disulfide bond formation (present or missing) was extracted from corresponding PDB structure. The B factor for each disulfide bond was calculated as the average of the 12 atoms per cysteine pair. To compare the B factor of present and missing disulfide bonds, the average disulfide bond B factors for all redundant structures were further averaged, respectively. There was no significant difference between the B factor of present (37.58 ± 21.78, s.d.) and missing (36.24 ± 21.02, s.d.) disulfide bonds (*p* = 0.0736, paired *t*-test).

### 3.1. Structural and functional features of the labile disulfide bonds

**Figure 1.**
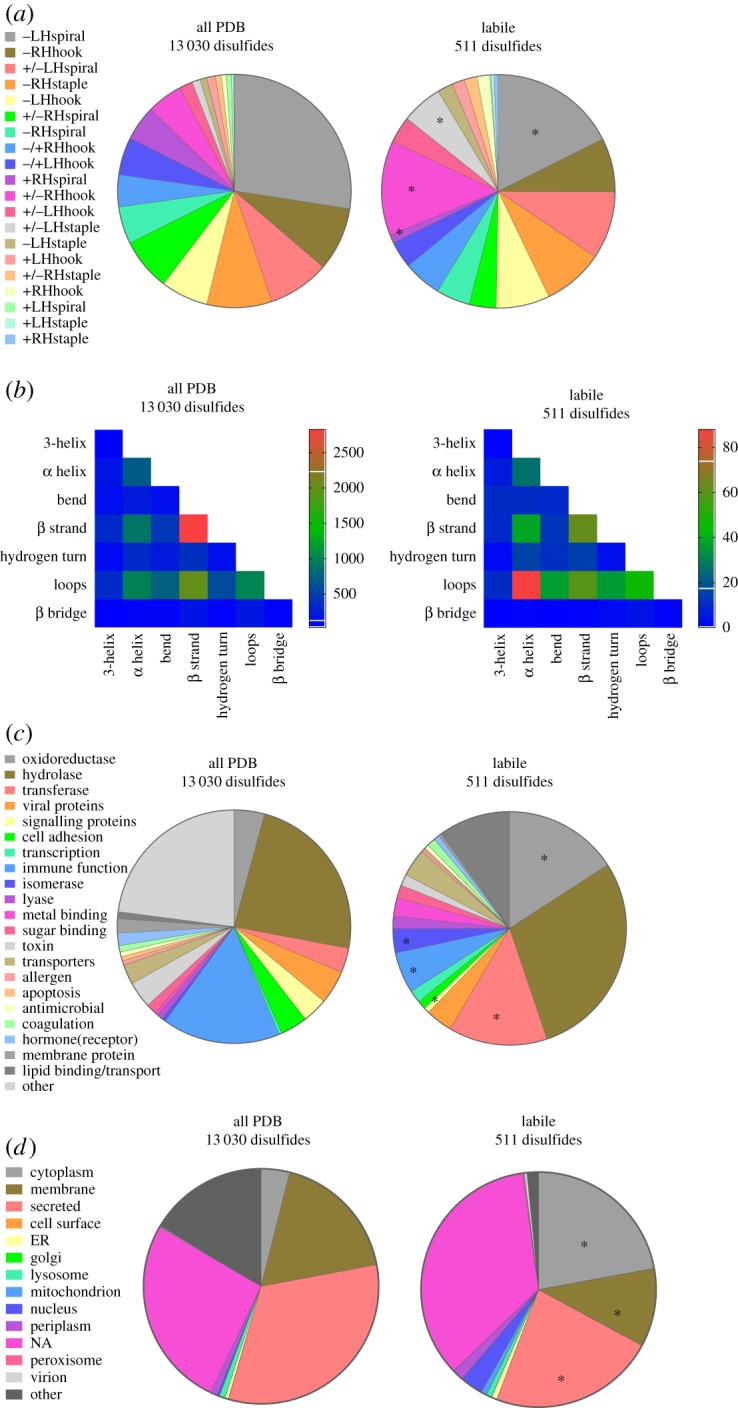
Structural and functional features of the labile disulfide bonds. (*a*) Distribution of the 20 disulfide bond configurations in unique disulfide bonds in PDB protein X-ray structures (13 030 disulfides, electronic supplementary material, table S1) and in labile bonds (511 disulfides, electronic supplementary material, table S2). Compared with the total PDB, the labile bonds are enriched in +/–RHhook and +/–LHstaple bonds (*χ*^2^ test, *p* < 0.0001) and have relatively fewer –LHspiral and +RHspiral bonds (*χ*^2^ test, *p* < 0.0001) (indicated by *). (*b*) Heatmap displaying the frequency of the secondary structures linked by disulfide bonds in all PDB protein structures and by labile disulfide bonds. There is enrichment of disulfides linking α-helices and loops in labile disulfide bonds (*χ*^2^ test, *p* < 0.0001). (*c*) Distribution of the functional classification of all proteins containing disulfide bonds and proteins containing labile disulfide bonds. Compared to the total PDB, there was a significant increase in oxidoreductases, transferases and isomerases (indicated by *). A significant decrease in disulfide bonds in proteins involved in signalling and immune function was observed (*χ*^2^ test, *p* < 0.0001). (*d*) Subcellular localization of all proteins containing disulfide bonds and proteins containing labile disulfide bonds. Compared to the total PDB, a significant increase in cytoplasmic proteins, as well as a decrease in membrane associated and secreted proteins was observed (*χ*^2^ test, *p* < 0.0001) (indicated by *).

By comparing the distribution of the 20 disulfide configurations between the entire PDB and labile disulfides, two differences were notable (figure 1*a*). Within the labile disulfide bonds, an increase in the +/–RHhook and +/–LHstaple configurations was observed (*χ*^2^ test, *p* < 0.0001). The –LHspiral, which is the main structural disulfide, as well as the +RHspiral configurations were decreased in the labile disulfides compared with the total PDB (*χ*^2^ test, *p* < 0.0001). The +/–RHhook is the predominant configuration of the catalytic disulfide bonds of oxidoreductases [7], such as PDI. This reflects the conserved position of this bond at the end of an α-helix in a thioredoxin fold. The catalytic disulfides of oxidoreductases undergo cycles of reduction and oxidation and there are several examples of oxidized and reduced structures in the PDB, hence their prevalence in the labile disulfide dataset. Of the 67 proteins in the labile disulfide dataset that have a +/–RHhook configuration, 22 are oxidoreductases (electronic supplementary material, table S2).

The secondary structures that a disulfide links can be informative. For instance, allosteric –RHstaple bonds often link adjacent strands in the same antiparallel β-sheet or constrain β-loops [4,16]. Also, the catalytic disulfide bonds of oxidoreductases link an α-helix to another or a loop structure. For the total PDB, disulfide bonds linking two β-strands were the most common, followed by linking of *β* strands and loops (figure 1*b*). For labile disulfides, enrichment of bonds linking α-helices and loops was observed (*χ*^2^ test, *p* < 0.0001) (figure 1*b*), which reflects the higher relative number of catalytic +/–RHhook configurations.

As anticipated, oxidoreductases were enriched in proteins containing labile disulfide bonds (figure 1*c*). Transferases, which includes kinases, methyltransferases and other enzymes that transfer functional groups, were also enriched in proteins containing labile disulfide bonds (figure 1*c*). Hydrolases and proteins involved in immune function were the largest category of disulfide-containing proteins in the PDB. Immune function proteins contain relatively fewer labile disulfides in this analysis. Overall, labile disulfide bonds were found in proteins of diverse functionalities.

The subcellular localization of proteins containing disulfide bonds was examined using the UniProt designation of the protein. A high proportion of cytoplasmic and nuclear proteins contained labile disulfide bonds (figure 1*d*). The cytoplasm and nucleus are environments traditionally thought not to be conducive to disulfide bond formation. This is not the case, however, as 509 disulfide bonds have been structurally defined in cytoplasmic proteins (electronic supplementary material, table S1). A high proportion of these disulfides (113, electronic supplementary material, table S2) have been characterized in oxidized and reduced states, indicating that they are unusually labile.

### 3.2. The labile disulfides are characterized by high strain

The conformational constraints on allosteric disulfides imposed by secondary structural features stress the bonds. The stresses fine tune their cleavage and thus the function of the protein. The stresses of the labile disulfides have been compared and contrasted with the average disulfide. There are different measures of disulfide bond stress [7,8].

**Figure 2.**
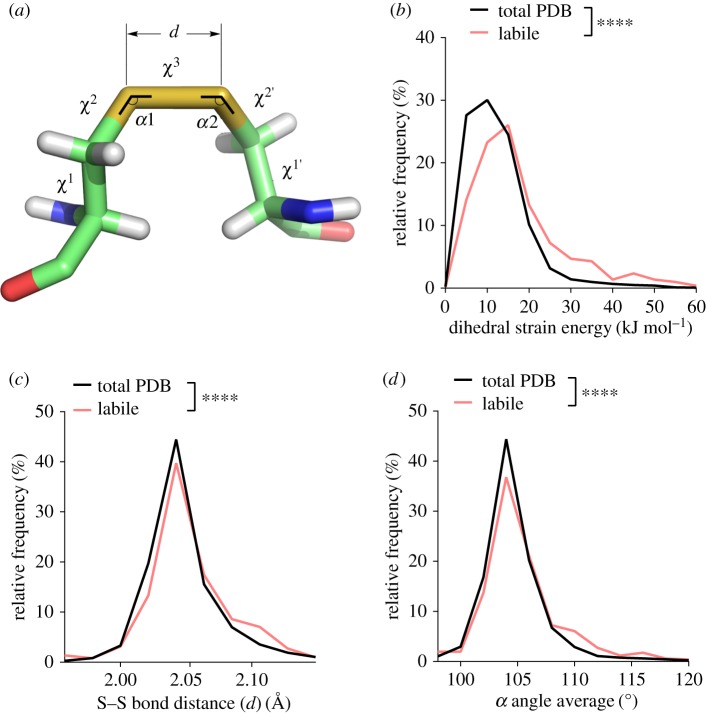
The labile disulfides are characterized by high dihedral strain energy, elongation of the sulfur–sulfur bond distance and stretching of the neighbouring bond angles. (*a*) Angles and distances of the cystine residue. The values *α*1 and *α*2 represent the two relevant bending angles of the disulfide, and the five dihedral angles are *χ*^1^, *χ*^2^, *χ*^3^, *χ*^2′^ and *χ*^1′^. *d* is the sulfur–sulfur bond length. (*b*) Relative frequency of DSE ranging from 0 to 60 kJ mol^−1^. The DSE was significantly increased for labile disulfides compared to all disulfide bonds in the PDB (*p* < 0.0001). (*c*) The relative frequency of the sulfur–sulfur bond distance ranging from 1.96 to 2.14 Å is shown. An increase in sulfur–sulfur bond distance is observed for labile disulfide bonds (*p* < 0.0001). (*d*) The average of both *α* angles was calculated for each disulfide bond. Shown is the relative frequency of the average angle ranging from 95 to 120°. Angles are increased for labile disulfide bonds (*p* < 0.0001). *T*-tests were used to compare total PDB to labile disulfides.

Dihedral strain energy (DSE) is an indicator of bond strain. The DSE is defined in terms of the torsion of the five dihedral or *χ* angles (figure 2*a*) of the cystine residue [17,18], and has been shown experimentally to reflect the amount of strain in a disulfide bond [19–22]. The length of the sulfur–sulfur bond and magnitude of the neighbouring angles (figure 2*a*) also reflect the stress in a disulfide [8]. The allosteric −RHstaple and −/+RHhook disulfide configurations carry tensile pre-stress in the bond due to direct stretching of the sulfur–sulfur bond and *α* angles, rather than by dihedral angle torsions [8]. This was shown using force distribution analysis, a technique for calculating atom–atom and residue–residue forces from molecular-dynamics simulations. As mechanical stretching of sulfur–sulfur bonds increases their redox potential [9–12], the pre-stressed bonds are more susceptible to cleavage.

The mean DSE of labile disulfide bonds was significantly higher than that of all disulfide bonds in the PDB (figure 2*b*). The mean DSE for all disulfide bonds was 12.48 kJ mol^−1^, whereas that of labile disulfide bonds was 17.64 kJ mol^−1^. The mean sulfur–sulfur bond length (figure 2*c*) and average *α* angle magnitude (figure 2*d*) were also significantly higher than those for all disulfide bonds. The mean sulfur–sulfur bond length of all disulfides was 2.046 Å, whereas for the labile disulfide bonds it was 2.055 Å. While the increased bond length is small at approximately 1 pm, the high stiffness of sulfur–sulfur bonds means that this change can entail substantial stress. The mean *α* angle of all disulfide bonds was 104.7°, whereas for the labile disulfide bonds it was 106.1°. Between 1 and 2° of stretching is also seen for the allosteric −RHstaple and −/+RHhook disulfide configurations [8]. Thus, the labile disulfide bonds are more stressed than the average bond based on three measures of strain.

Correlations between the measures of strain on disulfides were examined (electronic supplementary material, figure S2). DSE positively correlated with stretching of the *α* angles for both labile (*p* < 0.0001) and total (*p* < 0.0001) disulfides. There was no correlation between DSE and sulfur–sulfur bond length, or between sulfur–sulfur bond length and *α* angles for both labile and total disulfides. This indicates that stretching of the *α* angles is associated with high overall torsional strain, whereas sulfur–sulfur bond length is independent of the *α* angles.

### 3.3. The labile disulfides with allosteric configurations have higher dihedral strain

The allosteric –RHstaple, –LHhook and –/+RHhook configurations represented 9%, 6.5% and 4.5% of all disulfide bonds in the PDB (electronic supplementary material, figure S3*a*). For those allosteric disulfide bonds where there are high resolution crystal structures (*n* = 29, electronic supplementary material, table S3), these percentages increased to 39%, 16% and 16%, respectively (electronic supplementary material, figure S3*a*). The labile disulfides with allosteric configurations had a higher mean DSE than for all disulfide bonds (18.11 versus 12.74 kJ mol^−1^, *p* < 0.0001, *t*-test) (electronic supplementary material, figure S3*b*), which is consistent with the known properties of two (−RHstaple and −/+RHhook) of the three allosteric configurations.

### 3.4. Labile disulfide bonds are enriched in certain biological pathways

Five known allosteric disulfides were among the 511 labile bonds, which is a validation of this approach for identifying functional disulfides. These are the –RHstaple bonds in methionine aminopeptidase 2 [23], botulinum neurotoxins [24] and transglutaminase 2 [25], the –/+RHhook disulfides in plasminogen [26] and Lon protease [27] and the –LHhook bonds in DNA repair protein XRCC1 [28] and plasminogen [26] (electronic supplementary material, table S3).

To determine whether specific biological processes or pathways are enriched among human labile disulfides, the labile dataset was analysed using the DAVID Functional Annotation Tool [14,15]. The coagulation and complement cascades and oxygen-sensing HIF-1 pathway are significantly enriched in proteins containing labile disulfide bonds.

#### 3.4.1. Coagulation and complement cascades

**Figure 3.**
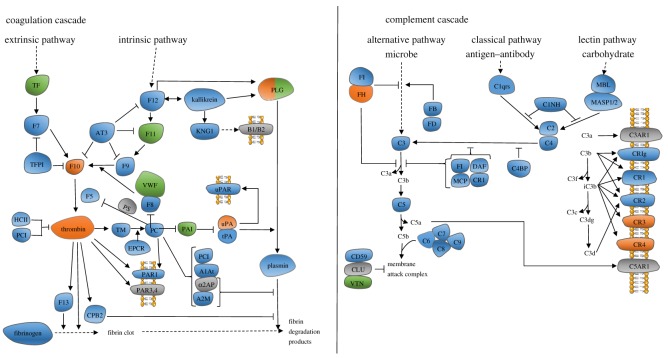
The coagulation and complement pathways are enriched in proteins containing labile disulfide bonds. The proteins containing labile disulfide bonds are shown in orange. The proteins shown in green contain characterized allosteric disulfide bonds (electronic supplementary material, table S3). Proteins shown in grey do not have any X-ray structures available in the PDB. FH: factor H. FI: complement factor I. FB: complement factor B. FD: complement factor D. C1NH: serpin family G member 1. C3: complement factor 3. C5: complement factor 5. DAF: CD55. MCP: CD46. CR1: complement C3b/C4b receptor 1. C1qrs: complement C1. MBL: mannose-binding lectin. MASP1/2: mannan-binding lectin serine peptidase 1/2. C2: complement C2. C4: complement C4. C4BP: complement component 4 binding protein. C5: complement C5. C6: complement C6. C7: complement C7. C8: complement C8. C9: complement C9. CLU: clusterin. VTN: vitronectin. C3AR1: complement C3a receptor 1. CRIg: V-set and immunoglobulin domain-containing protein 4. CR1: complement C3b/C4b receptor 1. CR2: complement C3d receptor 2. CR3: coagulation factor X/plasminogen. CR4: coagulation factor X/plasminogen. C5AR1: complement C5A receptor.

When the endothelium that lines blood vessels is damaged the process of thrombosis ensures that any leak is plugged and the endothelium is repaired. Thrombus formation relies on the deposition of two main components: platelets and the product of blood coagulation, fibrin. The complement system clears microbes and damaged cells from the circulation. The coagulation and complement pathways were enriched in proteins containing labile disulfide bonds (*p* = 0.0081, DAVID Functional Annotation Tool, KEGG pathway analysis, electronic supplementary material, table S2) [14,15] (table 1 and figure 3). Two proteins containing labile disulfide bonds are highlighted.

**Table 1.** Labile disulfide bonds in coagulation proteins. The first four characters are the PDB identifier followed by the strand. Underlined are the PDB structures used in figure 4.

protein	disulfide Cys	PDB with bond	PDB missing bond	configuration
coagulation factor X	168–182	KQCA^a^	3CENA, 2P95A	+/–RHspiral
urokinase plasminogen activator	11–19	3BT2A, 2I9AA, 2I9AC, 2I9AD	2FD6A	–/+LHhook
	50–111	2R2WU^b^	4X1NU, 4H42U, 4ZHLU, 4ZKSU, 1OWEA, 1OWHA	–RHstaple
	136–201	2R2WU^c^	4ZHLU	–RHstaple
plasminogen	481–560	5HPGA, 5HPGB, 4DURA	4DURB	–/+RHhook

^a^2W3KA3, 2VH6A, 3M37A, 2XBVA, 3KL6A, 3K9XB, 3K9XD, 2WYGA, 2Y80A, 4Y7AA, 2J34A, 3HPTB, 3HPTD, 2CJIA, 1NFYA, 2UWPA, 5K0HA, 3TK6A, 2Y81A, 2H9EH, 2RA0A, 1NFWA, 2EI7A, 2Y82A, 2Y5FA, 3M36A, 1MQ5A, 4Y7BA, 1F0SA, 2PHBA, 4BTUB, 4BTUF, 2P94A, 3LIWA, 2Y5HA, 2Y5GA, 2XC0A, 1XKAC, 1FJSA, 3CS7A, 2Y7XA, 2UWOA, 2UWOA, 3FFGA, 2FZZA, 2J4IA, 2J95A, 1WU1A, 4Y76A, 2D1JA, 2VWOA, 2VWNA, 1LPKB, 3Q3KA, 2VWLA, 2VWMA, 2VWMB, 4Y71A, 2P3UB, 2PR3A, 2P93A, 2W3IA, 2P16A, 3TK5A, 4ZHAA, 4BTIB, 4BTIF, 2Q1JA, 3KQEA, 2EI6A, 2Y7ZA, 3SW2B, 2G00A, 2P3TB, 1LPGB, 1LPZB, 3KQBA, 1NFUA, 3ENSB, 3ENSD, 1NFXA, 2VVVA, 1G2LA, 4A7IB, 2BOHB, 2JKHA, 1EZQA, 2J94A, 2BOKA, 2UWLA, 3IITA, 1KSNA, 1F0RA, 2XBWA, 2WYJA, 2VVCA, 2VVCB, 2VVUA, 2XC4A, 2J2UA, 1V3XA, 1Z6EA, 2XBYA, 1HCGA, 2XBXA, 4Y6DA, 2BQ7B, 2J38A, 2XC5A, 4Y79A, 2VH0A, 4ZH8A, 1XKBC, 1XKBD, 1MQ6A, 1C5MD, 2W26A, 2EI8A.

^b^4FU9A, 3OY5U, 3IG6B, 1W11U, 3KGPA, 2O8UA, 1W10U, 1W0ZU, 3OX7U, 3M61U, 4JNIU, 4OS1A, 1SQAA, 4FUEA, 1GJ7B, 2VIQA, 1U6QA, 1GJAB, 4FUGA, 1C5ZB, 3MWIU, 4OS6A, 1W12U, 4FUIA, 2VIVA, 4FUCA, 1EJNA, 4FU8A, 1VJAU, 1GJ9B, 4JK5A, 2VIPA, 3OY6U, 1SQOA, 4MNXA, 1GJDB, 1F5LA, 1SC8U, 1SQTA, 2VIWA, 4X0WU, 1W14U, 4OS2A, 4FUBA, 2O8WA, 1GI7B, 1O5BB, 1GJ8B, 4FU7A, 4OS4A, 4MNVA, 3MHWU, 3PB1E, 4ZKNU, 1LMWB, 1LMWD, 4MNYA, 4MNYB, 4X1RU, 1W13U, 4X1QU, 1VJ9U, 4ZHMU, 1GJBB, 3KHVA, 2VIOA, 1GJCB, 4FUHA, 2NWNA, 1GI8B, 1OWDA, 4JNLU, 4ZKRU, 2VNTA, 2VNTB, 2VNTC, 2VNTD, 2VNTE, 2VNTF, 2VINA, 1O5AB, 1GI9B, 4ZKOU, 1C5WB, 4X1SU, 4FUFA, 2O8TA, 4OS5A, 1C5XB, 1O5CB, 4X1PU, 4JK6A, 4OS7A, 3QN7A, 1O3PB, 4XSKU, 4FUJA, 1F5KU, 5HGGA, 5HGGB, 4FUDA, 4DVAU, 4MNWA, 4GLYA, 1C5YB.

^c^4FU9A, 3OY5U, 3IG6B, 1W11U, 4X1NU, 3KGPA, 4H42U, 2O8UA, 1W10U, 1W0ZU, 3OX7U, 3M61U, 4JNIU, 4OS1A, 1SQAA, 4FUEA, 1GJ7B, 2VIQA, 1U6QA, 1GJAB, 4FUGA, 1C5ZB, 3MWIU, 4OS6A, 1W12U, 4FUIA, 2VIVA, 4FUCA, 1EJNA, 4FU8A, 1VJAU, 1GJ9B, 4JK5A, 2VIPA, 3OY6U, 1SQOA, 4MNXA, 1GJDB, 1F5LA, 1SC8U, 1SQTA, 2VIWA, 4X0WU, 1W14U, 4ZKSU, 4OS2A, 4FUBA, 2O8WA, 1GI7B, 1O5BB, 1GJ8B, 4FU7A, 4OS4A, 4MNVA, 3MHWU, 3PB1E, 4ZKNU, 1LMWB, 1LMWD, 4MNYA, 4MNYB, 4X1RU, 1W13U, 4X1QU, 1VJ9U, 4ZHMU, 1GJBB, 3KHVA, 2VIOA, 1GJCB, 1OWEA, 4FUHA, 2NWNA, 1GI8B, 1OWDA, 4JNLU, 4ZKRU, 2VNTA, 2VNTB, 2VNTC, 2VNTD, 2VNTE, 2VNTF, 2VINA, 1O5AB, 1GI9B, 4ZKOU, 1C5WB, 4X1SU, 4FUFA, 2O8TA, 4OS5A, 1C5XB, 1O5CB, 4X1PU, 4JK6A, 4OS7A, 3QN7A, 1O3PB, 4XSKU, 4FUJA, 1F5KU, 5HGGA, 5HGGB, 4FUDA, 4DVAU, 4MNWA, 1OWHA, 4GLYA, 1C5YB.

#### 3.4.2. Urokinase-type plasminogen activator

**Figure 4.**
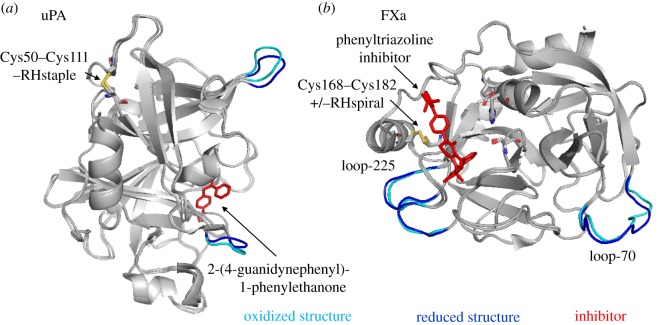
Conformational changes in proteins of the coagulation and complement pathways upon cleavage of the labile disulfide bonds. Structures of oxidized (cyan) and reduced (blue) proteins are aligned. Labile disulfides and their configurations are shown. The PDB identifiers are indicated in table 1. (*a*) uPA is shown in complex with the inhibitor, 2-(4-guanidynephenyl)-1-phenylethanone. The redox state of the Cys50–Cys111 disulfide influences the positions of loops surrounding the active site. (*b*) Factor Xa is shown in the presence of a phenyltriazoline inhibitor. The catalytic triad consisting of His57, Asp102 and Ser195 is shown in stick presentation. The redox state of Cys168–Cys182 influences slight conformational changes in Ca^2+^-binding loop-70 and Na^2+^-binding loop-225.

Urokinase-type plasminogen activator (uPA) is a serine proteinase that converts the zymogen plasminogen into the active protease plasmin. It consists of a serine protease, kringle and growth factor domain. A labile disulfide bond was found linking Cys50 and Cys111 in the kringle domain of uPA. Cleavage of this disulfide displaces the N-terminal charged loop that is implicated in uPA binding to its inhibitor, plasminogen activator inhibitor-1 (PAI-1) [29] (figure 4*a*). Replacing Cys11 with Tyr reduces uPA activity to 7% of wild-type protein [30].

#### 3.4.3. Factor Xa

Factor X (FX) is a vitamin K-dependent plasma zymogen that plays an essential role in blood coagulation. FX is activated through the extrinsic or intrinsic coagulation pathway by the tissue factor–VIIa complex or the IXa–VIIa complex, respectively. Activated FX (FXa) is then able to form the prothrombinase complex in association with factor Va, which cleaves prothrombin to form active thrombin on negatively charged phospholipid surfaces in the presence of calcium. A labile disulfide bond occurs in the catalytic domain of FXa, formed between Cys168 and Cys182 (chymotrypsin numbering). Slights shifts in loops outside of the catalytic domain of FXa are observed in reduced versus oxidized structures (figure 4*b*). Loop-70 forms a single Ca^2+^-binding site [31], whereas loop-225 is a Na^2+^-binding region [32]. Ca^2+^ and Na^2+^ binding both increase the catalytic efficiency of FXa, and thus thrombin generation, in a synergistic manner [33].

#### 3.4.4. Hypoxia inducible factor-1 signalling pathway

**Figure 5.**
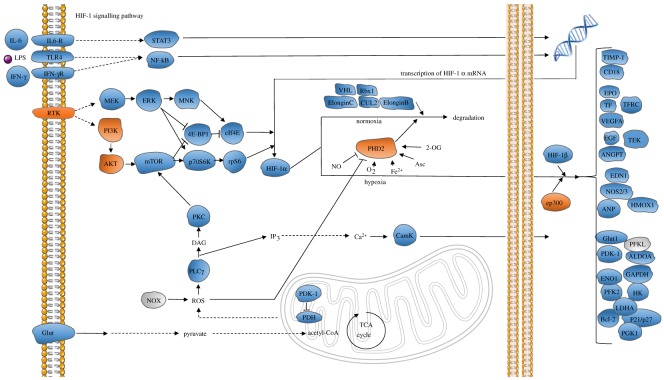
The HIF-1 signalling pathway is enriched in proteins containing labile disulfide bonds. The proteins containing labile disulfide bonds are shown in orange. Grey indicates that no X-ray structures are available of this protein. RTK: epidermal growth factor receptor/erb-b2 receptor tyrosine kinase. MEK: mitogen-activated protein kinase ½. ERK: mitogen-activated protein kinase 1/3. MNK: MAP kinase interacting kinase ½. STAT3: signal transducer and activator of transcription 3. 4E-BP1: eukaryotic translation initiation factor 4E-binding protein. eIf4E: eukaryotic translation initiation factor 4E. p70S6 K: ribosomal protein S6 kinase. Rps6: ribosomal protein S6. VHL: Von Hipper Lindau tumor suppressor. RBX1: ring box 1. CUL2: cullin 2. PI3 k: phosphoinositide-3-kinase. AKT: protein kinase B. mTOR: mechanistic target of rapamycin. ElonginC: transcription elongation factor B subunit 1. ElonginB: transcription elongation factor B subunit 2. PHD2: Egl-9 family hypoxia inducible factor 1. CamK: calcium/calmodium-dependent protein kinase. ep300: E1A-binding protein p300. PLCɣ: phospholipase C gamma. NOX: NADPH oxidase. Glut: solute carrier family 2 member 1. TIMP-1: TIMP metallopeptidase inhibitor 1. CD18: lymphotoxin beta receptor. EPO: erythropoietin. TF: transferrin. TFRC: transferrin receptor. VEGF: vascular endothelial growth factor. Flt-1: Fms-related tyrosine kinase 1. EGF: epidermal growth factor. TEK tyrosine Kinase: TIE-2, angiopoietin-1 receptor. EDN1: endothelin 1. NOS2/3: nitric oxide synthase 2/3. ANP: natriuretic peptide A. PDK-1: pyruvate dehydrogenase kinase 1. HK: hexokinase. PFKL: ATP-dependent 6-phosphofructokinase. GAPDH: glyceraldehyde-3-phosphate dehydrogenase. ALDOA: fructose-bisphosphate aldolase A. ENO1: enolase 1. PGK1: phosphoglycerate kinase 1. PFK2: 6-phosphofructo-2-kinase/fructose-2,6-biphosphatase 3. LDHA: l-lactate dehydrogenase A chain. Bcl-2: apoptosis regulator Bcl-2. p21/p27: cyclin-dependent kinase inhibitor 1.

Low oxygen levels lead to the activation of the HIF-1 pathway, which is conserved across metazoan species. Activation of this pathway results in rapid accumulation of the transcription factor, HIF-1*α*, which stimulates expression of glycolysis genes in response to suppressed oxidative phosphorylation [34,35]. The HIF-1 signalling pathway is enriched in human proteins containing labile disulfide bonds (*p* = 0.026, DAVID Functional Annotation Tool, KEGG pathway analysis, electronic supplementary material, table S2) (figure 5). Three of these proteins are described below. All HIF-1 pathway proteins containing labile disulfide bonds are shown in table 2.

**Table 2.** Labile disulfide bonds in the HIF-1 signalling pathway. The first four characters are the PDB identifier followed by the strand. Underlined are the PDB structures used in figure 6.

protein	disulfide Cys	PDB with bond	PDB missing bond	configuration
AKT1	60–77	4EJNA, 2UZRA, 1UNPA, 1UNRA	1UNQA, 1H10A, 2UZSA, 2UVMA	–RHstaple
EP300	1796–1801	3P57P	3IO2A	+/–RHspiral
	1796–1806	3P57P	*3IO2A*	+LHspiral
Egl 9 homolog 1 (PHD2)	201–208	4BQYA, 3OUIA, 4BQXA, 4JZRA, 5LATA, 4BQWA, 2Y33A, 3HQRA, 5LBEA, 5LBFA, 2HBTA, 5LBBA, 5LB6A	4UWDA, 3HQUA, 4KBZA, 3OUJA, 2HBUA, 5LBCA, 2G19A, 2G1MA, 2Y34A	+/–LHspiral
EGFR	510–523	1MOXA, 1MOXB, 3P0YA, 4UV7A, 5SX5N	5SX5M	–RHstaple
PIK3CG	357–524	4FULA	4ANVA,3R7QA,5G55A	–LHstaple

#### 3.4.5. Protein kinase B (AKT1)

**Figure 6.**
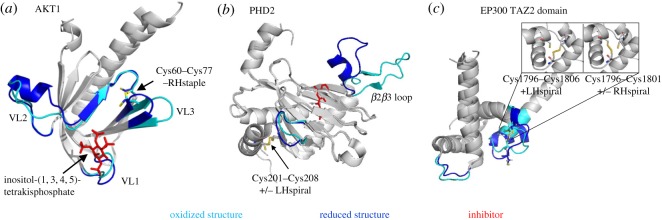
Conformational changes in HIF-1 pathway proteins upon cleavage of the labile disulfide bonds. Structures of oxidized (cyan) and reduced (blue) proteins are aligned. Labile disulfides and their configurations are shown. The PDB identifiers are indicated in table 2. (*a*) PH domain of AKT1 with bound phosphoinositol. The positions of variable loops (VL) 1, 2 and 3 differ in oxidized and reduced structures. (*b*) The position of the *β*2*β*3 loop of PHD2 differs in oxidized and reduced structures. (*c*) The positions of the zinc-binding loops connecting the α-helices in the TAZ2 domain of EP300 differ in oxidized and reduced structures. The insets depict the two possibilities of disulfide bonding between the three participating cysteines within the oxidized structure.

AKT1 is a serine/threonine kinase that plays a central role in glycogen metabolism, cell survival, proliferation and angiogenesis. Cytokines and growth stimuli activate phosphoinositide 3-kinase to generate phosphatidylinositol (3,4,5)-triphosphate (PIP3) patches at the plasma membrane. AKT1 resides in the cytosol but translocates to plasma membrane through interaction with PIP3 via its N-terminal pleckstrin-homology (PH) domain. AKT1 is then activated by phosphoinositide-dependent kinase I and mechanistic target of rapamycin complex by phosphorylation at Thr308 and Ser473, respectively. The PH domain contains a labile disulfide bond that links cysteines 60 and 77 (figure 6*a*). Notably, this bond is formed in all apo structures of the PH domain (e.g. PDB identifier 1UNP) but absent in all structures of the domain bound to inositol phospholipids (e.g. PDB identifier 1UNQ). In addition, the Cys60–Cys77 disulfide has the archetypal –RHstaple allosteric configuration. Reduction of the disulfide bond results in movement of variable loop (VL) 3 adjacent to the disulfide bond and VL1 that lines the phosphoinositol binding pocket (figure 6*a*). Additionally, a short acidic *α* helix in VL2 is present when the disulfide is intact but not when the bond is cleaved. Cys60 and Cys77 are conserved in AKT2 and AKT3, as well as in mouse and rat AKT1.

#### 3.4.6. Prolyl hydroxylase-containing protein 2

Prolyl hydroxylase-containing protein 2 (PHD2) is encoded by the EGLN1 gene. PHD2 controls the activity of HIF-1*α* by hydroxylation, which leads to polyubiquitination and degradation of the transcription factor. As oxygen levels decrease, PHD2 fails to hydroxylate HIF-1*α* which then relocates to the nucleus to stimulate expression of glycolysis-related genes [35]. PHD2 contains a labile disulfide bond at Cys201–Cys208. Of the many crystal structures available in the PDB, about half of them show PHD2 with a reduced disulfide bond (table 2). The *β*2*β*3 loop encompassing residues 238–250 determines substrate specificity of PHD2 [36] and its position in the structure is influenced by the redox state of the Cys201–Cys208 bond (figure 6*b*). Notably, reactive oxygen species are implicated in control of PHD2 activity by mediating disulfide-linked homodimerization of the enzyme [37], leading to activation of the HIF-1 pathway and a switch from oxidative phosphorylation to glycolysis.

#### 3.4.7. EP300

EP300 is a histone acetyl transferase that is crucial for normal gene regulation. EP300 and the closely related CBP are involved in binding and coordinating the assembly of transcription factor complexes to influence gene transcription. EP300 consists of multiple well-defined domains, including TAZ1 and TAZ2 domains, which surround the catalytic core. The catalytic core consists of a bromodomain that recognizes acetylated substrates, a HAT domain that acetylates histones and proteins [38,39], and a CH2 region containing a RING domain [40]. A labile disulfide bond was identified in the TAZ2 domain of EP300 that mediates interaction with transcription factors and binds zinc (table 2). Interestingly, this bond can differentially form between two of three cysteines (see insets of figure 6*c*). The disulfide bond can link Cys1796 and Cys1806 or Cys1796 and Cys1801. The redox state of the disulfide influences the conformation of the loops linking the α-helices and may influence zinc binding (figure 6*c*). Zinc binding is increasingly recognized as being redox sensitive, with reactive oxygen species-mediated disulfide bond formation triggering the release of zinc ions [41].

## 4. Discussion

External mechanical forces regulate cleavage of protein disulfide bonds [9–12,42–46]. Rates of thiol/disulfide bond exchange are subject to mechano-chemical coupling. That is, the reactivity of a disulfide bond can be increased or decreased by mechanical forces that stretch, bend and twist the sulfur–sulfur and neighbouring bonds. For example, stretching of the sulfur–sulfur bond enhances cleavage of the disulfide.

Internal mechanical forces also control cleavage of protein disulfide bonds in an analogous fashion [8]. Two of the twenty disulfide bond configurations, the –RHstaple and –/+RHhook bonds, are particularly subject to topological stresses and allosteric function has been reported for seventeen of these bonds thus far. The –LHhook configuration is also associated with allosteric function with seven examples thus far, although these bonds are no more stressed than the other eighteen configurations and it remains to be determined the reason for this functional association.

While these biophysical properties are informative and have proved useful for identifying new allosteric bonds, it is likely that the majority of the approximately 2800 disulfide bonds with allosteric configurations in known protein structures will not be redox active. To facilitate identification of this post-translational control of protein function, we mined X-ray structures for labile disulfide bonds that exist is some structures of a protein but are reduced in others. Our hypothesis is that the facile nature indicates a propensity for cleavage and so possible allosteric regulation of the protein in which the disulfide resides.

The limitations of this analysis are the availability of crystal structures, potential differences in the qualities of the structures themselves, and the non-native conditions that may have been employed to obtain the crystals and structures. For example, purifying and crystallizing cytosolic proteins in oxidizing conditions. It is also possible that some of the identified labile bonds are the result of cleavage by X-rays during data collection [47], or inefficient formation during maturation of the protein. This does not exclude an allosteric function for these particular bonds, although they may have no functional role in the protein.

Five hundred and eleven labile disulfide bonds were identified and we suggest that these bonds are enriched in allosteric disulfides. This conclusion is supported by the finding that the labile bonds are stressed based on high average dihedral strain coupled with an average elongated sulfur–sulfur bond length and extended bond angles. As stretching of the sulfur–sulfur bond makes disulfides easier to cleave [9], this feature is likely a major reason why the identified bonds are labile. Five known allosteric disulfides were captured in the labile bonds and visual inspection of a number of the labile bonds suggested an allosteric function, which further supports the conclusion that the labile bonds are enriched in allosteric disulfides.

Blood coagulation and haemostasis are processes that are regulated by allosteric disulfide bonds [2]. Four different secreted oxidoreductases, PDI [48], ERp57 [49], ERp5 [50] and ERp72 [51], have been found to be essential for thrombosis in mice, and proteins involved in thrombosis, such as thrombospondin-1 [52], vitronectin [53], plasminogen [26] and tissue factor [54], are known to be regulated by allosteric disulfides. Notably, tissue factor expression [55] and activity [56] have been linked to complement activation. PDI and complement activation has also been linked to tissue factor decryption [57]. Anti-thymocyte globulin activates tissue factor on monocytes and PDI inhibitors block this activation. In addition, C5 complement activation on monocytes results in oxidation of cell surface PDI. It is perhaps not surprising, therefore, that the coagulation and complement cascades are enriched in labile disulfides. For instance, the Cys50–Cys111 disulfide in uPA may be a substrate for one or more of PDI, ERp57, ERp5 and ERp72. Cleavage of the bond could regulate its inactivation by PAI-1.

A high proportion of labile disulfide bonds occur in proteins that reside in the cytoplasm or nucleus. This finding implies that these intracellular compartments are conducive to this post-translational protein control. Cleavage and/or formation of the labile disulfides is presumably enabled by the precise redox buffering mechanisms of the cytoplasm/nucleus. Pathway analysis showed that proteins involved in the HIF-1 oxygen homeostasis system were enriched among human proteins containing labile disulfides. Notably, reversible disulfide bond formation has been speculated to be a common regulatory mechanism of oxygen sensors [41]. For example, a labile disulfide was identified in the histone acetyl transferase, EP300. The bond is in the TAZ2 domain that mediates interaction with oxygen-sensing HIF-1*α*. Cleavage and/or formation of this bond is predicted to influence HIF-1*α* binding.

## Supplementary Material

Supplementary material

## Supplementary Material

Table S1

## Supplementary Material

Table S2

## Data accessibility

Supporting data are available at Dryad (https://doi.org/10.5061/dryad.84827) [58].
