# Supplementary material for: Identification of allosteric disulfides from labile bonds in X-ray structures
Source: R Soc Open Sci. 2018 Feb 7;5(2):171058. doi: 10.1098/rsos.171058 (PMC5830721; doi:10.1098/rsos.171058)
Supplement: Supplementary material [file rsos171058supp1.pdf]

Supplementary Information

Identification of allosteric disulphides from labile bonds in X-ray structures

Aster E Pijning, Joyce Chiu, Reichelle X Yeo, Jason W H Wong, Philip J Hogg

Table S1. Excel spreadsheet of unique disulphides in a culled set of X-ray structures described by G. Wang and R. Dunbrack, Jr. (file pdbaanr).

Table S2. Excel spreadsheet of labile disulphide bonds present in some molecules of a protein crystal but absent in others (same PDB, sheet 1), or present in some structures of a protein but absent in others (different PDB, sheet 2).

Table S3. Allosteric disulphides with known configurations.

**Protein**	**Species**	**Disulphide Cys**	**PDB ID**	**Configuration**	**Reference**
Methionine aminopeptidase 2	*Homo sapiens*	228-448	1B59	–RHstaple	(Chiu et al., 2014)
Tissue Factor	*Homo sapiens*	186-209	1BOY	–RHstaple	(Chen et al., 2006)
Botulinum neurotoxins	*Clostridium botulinum*	436-445	1EPW	–RHstaple	(Swaminathan and Eswaramoorthy, 2000)
Prolyl cis-trans isomerase, AtFKBP13	*Arabidopsis thaliana*	106-111	1Y0O	–RHstaple	(Gopalan et al., 2004)
HIV gp120	*Human immunodeficiency virus type 1*	126-196	1YYM	–RHstaple	(Auwerx et al., 2009; Azimi et al., 2010; Barbouche et al., 2003; Gallina et al., 2002; Matthias et al., 2002; Ou and Silver, 2006; Pinkas et al., 2007; Reiser et al., 2012)
		296-331		–RHstaple	
		385-418		–RHstaple	
Transglutaminase 2	*Homo sapiens*	370-371	2Q3Z	–RHstaple	(Malojcic and Glockshuber, 2010)
CD4	*Homo sapiens*	130-159	3CD4	–RHstaple	(Wang et al., 2011)
Arylsulfate sulfotransferase	*Escherichia coli*	418-424	3ELQ	–RHstaple	(Zhou et al., 2014)
C-reactive protein	*Homo sapiens*	36-97	3L2Y	–RHstaple	(Ioannou et al., 2010)
von Willebrand factor	*Homo sapiens*	2451-2468	3BK3	–RHstaple	(Giannakopoulos et al., 2012)
β2-glycoprotein I	*Homo sapiens*	288-326	1C1Z	–/+RHhook	(Zhou et al., 2010)
Factor XI	*Homo sapiens*	362-482	2F83	–/+RHhook	(Nishii et al., 2015)
Angiotensinogen	*Homo sapiens*	18-138	2WXW	–/+RHhook	(Butera et al., 2013)

Lon protease[1]	*Homo sapiens*	617-691	3WU4	–/+RHhook	(Metcalfe et al., 2012)
Plasmin(ogen)	*Homo sapiens*	462-541	4DUR	–/+RHhook	(Kellett-Clarke et al., 2015)
Interleukin receptor subunit gamma	*Homo sapiens*	160-209	2ERJ	–LHhook	(Chiu et al., 2013)
CD44	*Mus musculus*	81-101	2JCP	–LHhook	(Cuneo and London, 2010)
Vascular endothelial growth factors C & D	*Homo sapiens*	156-165	2X1W	–LHhook	(Butera et al., 2013)
XRCC1	*Homo sapiens*	12-20	3LQC	–LHhook	(Mohammad et al., 2017)
Plasmin(ogen)	*Homo sapiens*	512-536	4DUR	–LHhook	(Sagong and Kim, 2017)
QueF	*Bacillus subtilis*	55-99	5UDG	–LHhook	(Liu and Cowburn, 2016)
CgDapF	*Corynebacterium glutamicum*	83-221	5H2G	–LHhook	(Pinkas et al., 2007)
C-terminal Src kinase[2]	*Homo sapiens*	122-164	3EAC	+LHhook	(Cook et al., 2013)
Transglutaminas e 2	*Homo sapiens*	230-370	3LY6	+RHstaple	(Kim et al., 2015)
hTryptaseβ[3]	*Homo sapiens*	191-220	2FPZ	–RHhook	(Kaiser et al., 2007)
Thiolase	*Clostridium acetobutylicum*	88-378	4XL2	+/–LHspiral	(Wilczynska et al., 2003)
MICA	*Homo sapiens*	202-259	1HYR	–/+LHhook	(Chiu et al., 2014)
PAI-2	*Homo sapiens*	79-161	1BY7	Undetermined	(Chen et al., 2006)

1. Also exists in a +/–LHspiral configuration (PDB ID 3WU4, chain A)
2. Also exists in a +/–RHspiral configuration (PDB ID 3EAC, chain A)
3. Also exists in a –RHspiral configuration (PDB ID 2FPZ, chain B)

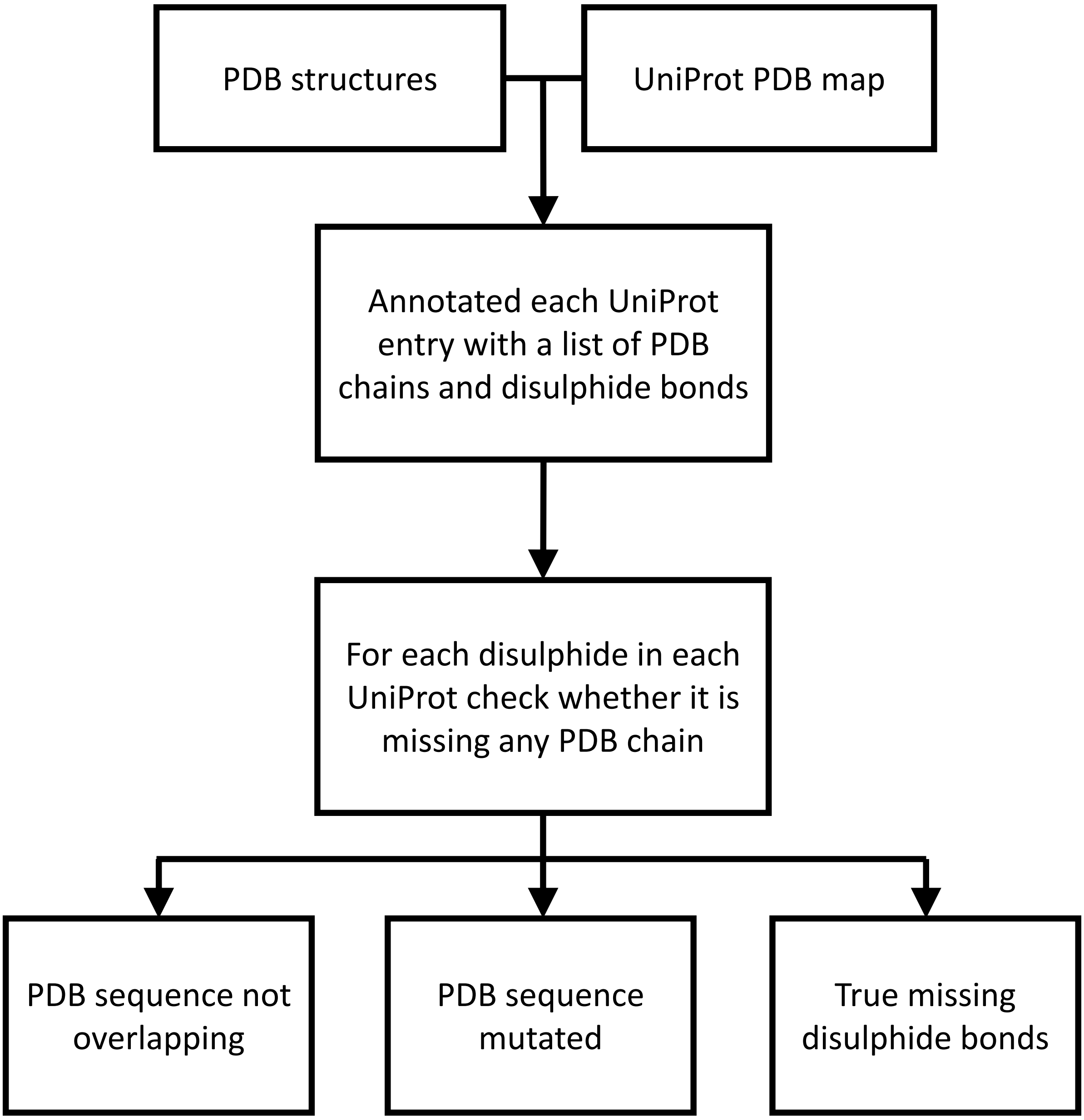

Figure S1. Flow chart for mining of PDB X-ray structures for labile disulphide bonds.

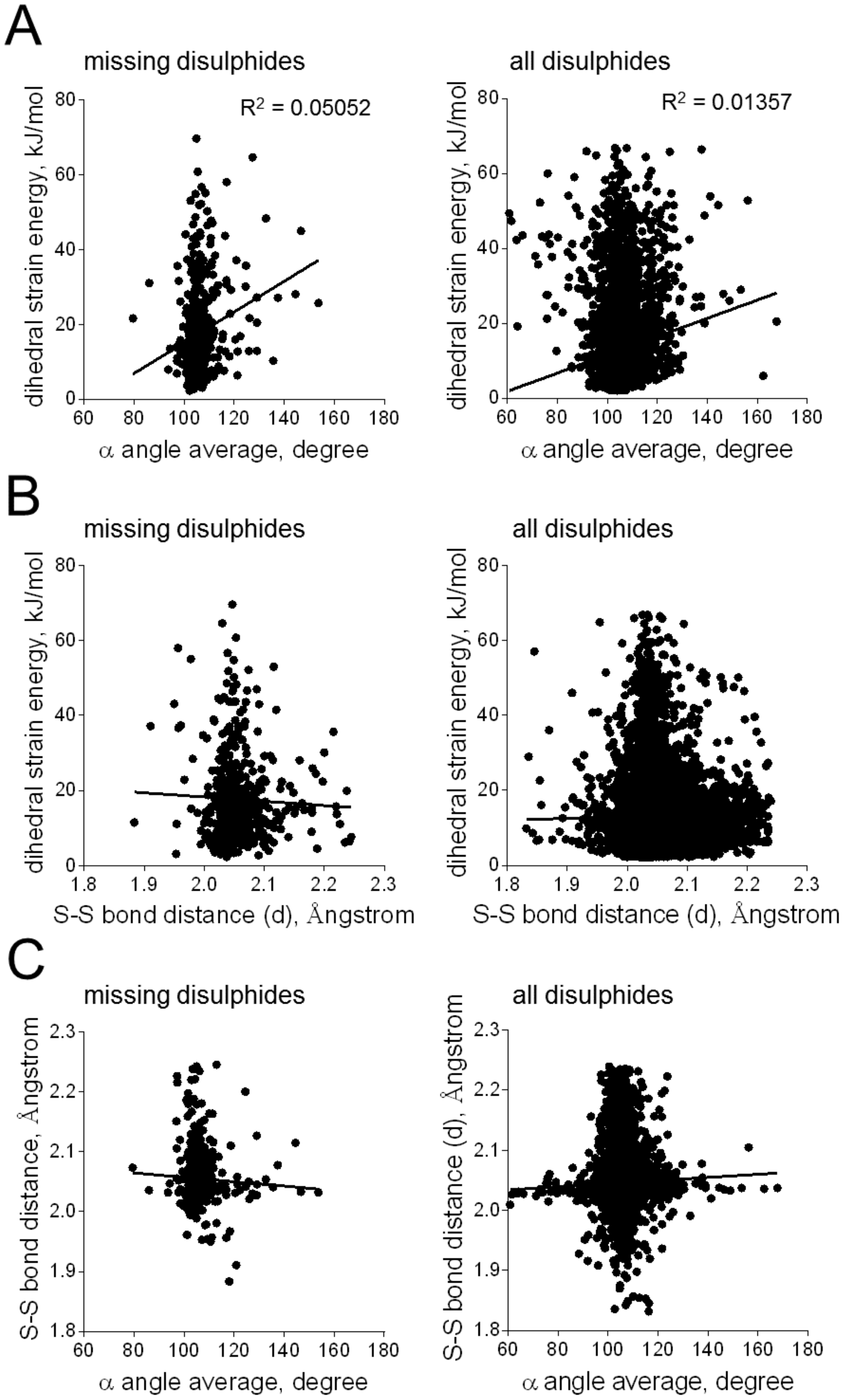

Figure S2. Correlations of the measures of strain of the missing and all disulphides. **A**. Positive correlation of DSE with stretching of the α angle for both missing and all disulphides. **B**. No correlation between DSE and length of the sulphur-sulphur bond of disulphides for both missing and all disulphides. **C**. No correlation between sulphur-sulphur bond length and α angle for both missing and all disulphides.

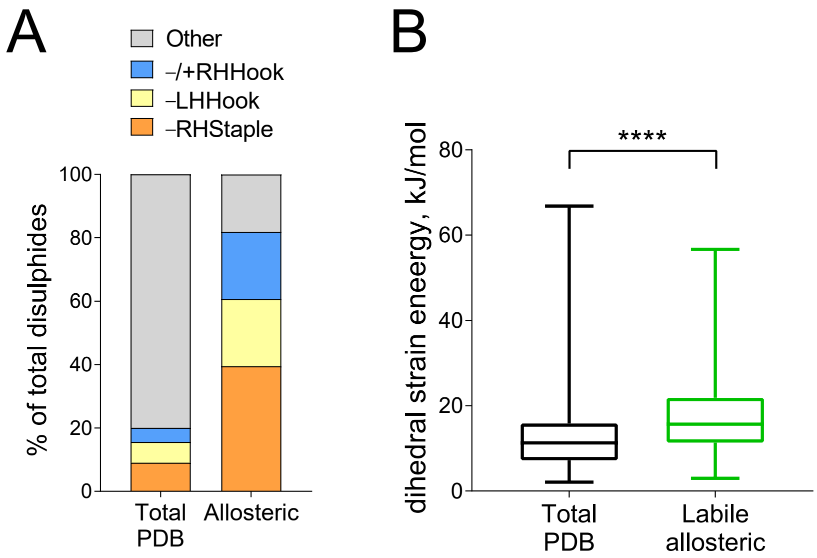

Figure S3. The labile disulphides with allosteric configurations have higher dihedral strain energy. **A**. The –RHStaple, –LHHook, and –/+RHHook configurations represent 9%, 6.5%, and 4.5% of all disulphide bonds in the PDB. For the structurally defined allosteric disulphide bonds (n = 29, S2 Table), these percentages increase to 39%, 16%, and 16%, respectively. **B**. The labile disulphide bonds with allosteric configurations (n = 158) have an increased DSE compared to the total PDB ($p<0.0001$, Mann-Whitney test).
